# Increasing the ionic conductivity and lithium-ion transport of photo-cross-linked polymer with hexagonal arranged porous film hybrids

**DOI:** 10.1016/j.isci.2022.104910

**Published:** 2022-08-13

**Authors:** Manjit Singh Grewal, Kazuaki Kisu, Shin-ichi Orimo, Hiroshi Yabu

**Affiliations:** 1 Advanced Institute of Materials Research (WPI-AIMR), Tohoku University, 2-1-1, Katahira, Aoba-Ku, Sendai 980-8577, Japan; 2 Institute for Materials Research (IMR), Tohoku University, Katahira 2-1-1, Aoba-ku, Sendai 980-8577, Japan; 3 Institute of Multidisciplinary Research for Advanced Materials (IMRAM), Tohoku University, 2-1-1, Katahira, Aoba-Ku, Sendai 980-8577, Japan; 4 RIKEN Center for Emergent Matter Science, 2-1, Hirosawa, Wako, Saitama 351-0198, Japan

**Keywords:** energy storage, materials science, polymers

## Abstract

High ionic conductivity, suitable mechanical strength, and electrochemical stability are the main requirements for high-performance poly(ethylene oxide)-based electrolytes. However, the low ionic conductivity owing to the crystallinity of the ethylene oxide chain that limits the discharge rate and low-temperature performance has restricted the development and commercialization of these electrolytes. Lithium electrolytes that combine high ionic conductivity with a high lithium transference number are rare and are essential for high-power batteries. Here, we report hexagonal arranged porous scaffolds for holding prototype polyethylene glycol-based composite electrolytes containing solvate ionic liquid. The appealing electrochemical and thermal properties indicate their potential as electrolytes for safer rechargeable lithium-ion batteries. The porous scaffolds in the composite electrolytes ensure better electrochemical performance towing to their shortened pores (sizes of 3-14 μm), interconnected pathways, and improved lithium mobility. We demonstrate that both molecular design and porous microstructures are essential for improving performance in polymer electrolytes.

## Highlights

• Robust HCPE films were fabricated using the breath-figure method
• HCPE shows high ionic conductivity of 6.66 × 10^−4^ S cm^−1^
• HCPE exhibits ESW of 4.7 V vs Li+/Li at 60°C and excellent compatibility with Li
• Molecular design and porous-microstructures are essential for electrolyte performance

## Graphical abstract

**Figure:**
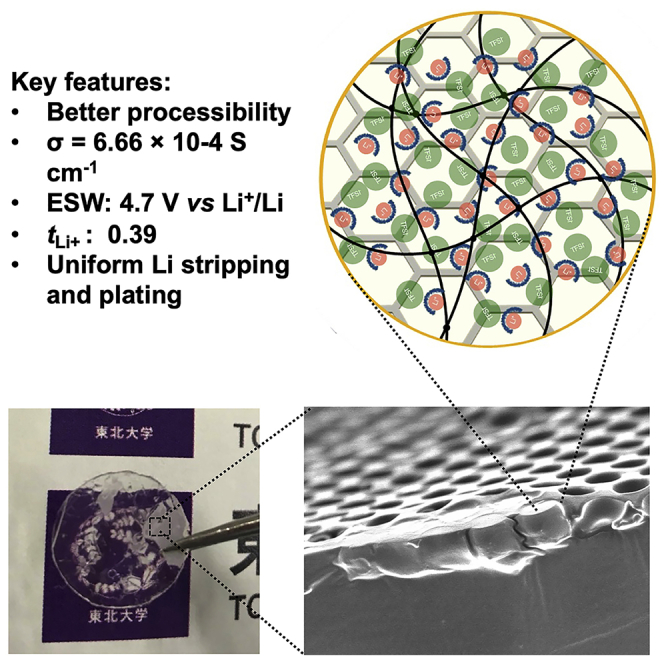

## Introduction

Lithium-ion batteries (LIBs) are important as power sources for many portable electrical devices (e.g., laptops and mobile phones) and the use of LIBs is expected to grow substantially and expand into new applications (Wu and Cui, 2012; Armand and Tarascon, 2008; Grewal et al., 2020a, 2020b). Poly(ethylene oxide) (PEO) is the most studied polymeric host for solid polymer electrolytes owing to its impressive solvating properties for a wide variety of salts through the interaction of its ether oxygen with lithium ions (Grewal, Tanaka, & Kawakami, 2019a, 2019b). However, there are two major problems with the electrolytes in PEO-based LIBs. One is the decrease in ionic conductivity at room temperature owing to the crystallization of PEO and the other is short circuits caused by lithium dendrites forming during the charging process (Grewal et al., 2019a, 2019b, *Polymer International*; Grewal et al., 2020a, 2020b; Bai et al., 2016, 2018). Cross-linking can reduce the crystallinity and increase mechanical stability (Lu et al., 2017; Suk et al., 2016; He et al., 2012). Good mechanical aspects (Stolz et al., 2021a, 2021b), ease of film fabrication with desirable shapes and sufficient thickness (Grewal et al., 2021, Stolz et al., 2021a, 2021b) are crucial to suppress dendrite penetration (Kasnatscheew et al., 2020) and short circuits; including minimizing the use of separators. In particular, cross-linking via UV irradiation is efficient because its energy efficiency is good, its polymerization time is short, its process control is easy, and it is a solvent-free method (Wei et al., 2018; Porcarelli et al., 2016). In the PEO-based electrolytes, by concurrent exploitation of photo-induced crosslinking and *in situ* functionalization procedures, the suppression of PEO chain crystallization is achievable at ambient conditions, resulting in polymer electrolytes that possess solid-like properties without hampering ionic mobility. Furthermore, the use of ionic liquids in cross-linked network polymers is promising for increasing ionic conductivity (MacFarlane et al., 2014; Armand et al., 2009; Kalhoff et al., 2015). An example of lithium-ion solvate ionic liquids (SILs), such as equimolar mixtures of organic ligand glymes (e.g., triglymes or tetraglymes) and lithium salts (e.g., lithium bis(trifluoromethanesulfonyl)imide (LiTFSI)), have attracted interest for their remarkable properties, including room temperature ionic conductivity of at least 1 mS cm^−1^, non-flammability, low vapor pressure, low melting point, and good thermal and chemical stabilities (Li et al., 2018; Grewal et al., 2021; D’Angelo and Panzer, 2017; Kido et al., 2015, Osada et al., 2016). These properties make them promising candidates as non-flammable, non-volatile, stable electrolytes for lithium-based energy storage devices.

However, SIL electrolytes have low lithium transference numbers owing to the small fraction of mobile lithium ions or owing to solvation when the mobility of anion is also higher in such electrolytes (Schaefer et al; 2013; Thomas et al., 2000) and still suffer from short circuits caused by unstable lithium growth formation (Bazant et al., 2016). Several approaches have been reported to address these problems, such as using gel-like films, creating semi-crystalline structures with amorphous domains, embedding fillers or inorganic nanoparticles (Croce et al., 1998), using electrospun polymers or block polymers, using ceramics, and coating with polymer matrices (Kitazawa et al., 2014; Unemoto et al., 2013; Saito et al., 2007; Snedden et al., 2003; Chereddy et al., 2020). Aliahmad et al., 2016 reported a porous PVDF-HFP membrane containing ionic liquid for improving ionic conductivity. Prasanth et al. (2013) prepared a PVDF-clay nanocomposite membrane with better electrochemical performance. Qin et al. (2020) fabricated a composite polymer electrolyte based on a cellulose nanofibril aerogel infiltrated with PEO and LiTFSI. However, most of these approaches have produced stiff materials with low ionic conductivity or have required complex fabrication procedures.

To address these problems, we prepared microporous polymer films with a hexagonal arrangement of pores to contain electrolytes. Hexagonal-patterned porous (HCP) films that mimic the natural honeycomb structure are useful scaffold materials owing to their light weight, mechanical strength, and large surface area (Male and Huh, 2019; Yabu, 2018). Previously, we have reported the fabrication of HCP films using the breath-figure technique (Nakamichi et al., 2011). By casting a polymer solution under humid conditions, water droplets condense on the surface of the polymer solution, and then are close-packed by capillary force. After complete evaporation of the solvent and template water droplets, HCP films are formed (Yabu et al., 2006; Ishii et al., 2009). HCP films have been used in various applications, such as filtration (Chen et al., 2022), controllable oil adhesion (Chen et al., 2020), liquid repellent surfaces (Kamei and Yabu, 2015; Yabu and Shimomura, 2005), cell culture substrates (Kawano et al., 2014; Oku et al., 2021)-34], and capacitors (Shimamura et al., 2019). However, the potential of hexagonal arranged porous architectures for LIB electrolytes has not been explored. Membranes with small pores can provide steric barriers or increase internal resistance, hindering ion transport, whereas membranes with pores that are too large can induce internal short circuits, causing LIB failure (Kotobuki et al., 2010; Kang et al., 2020).

**Figure 1:**
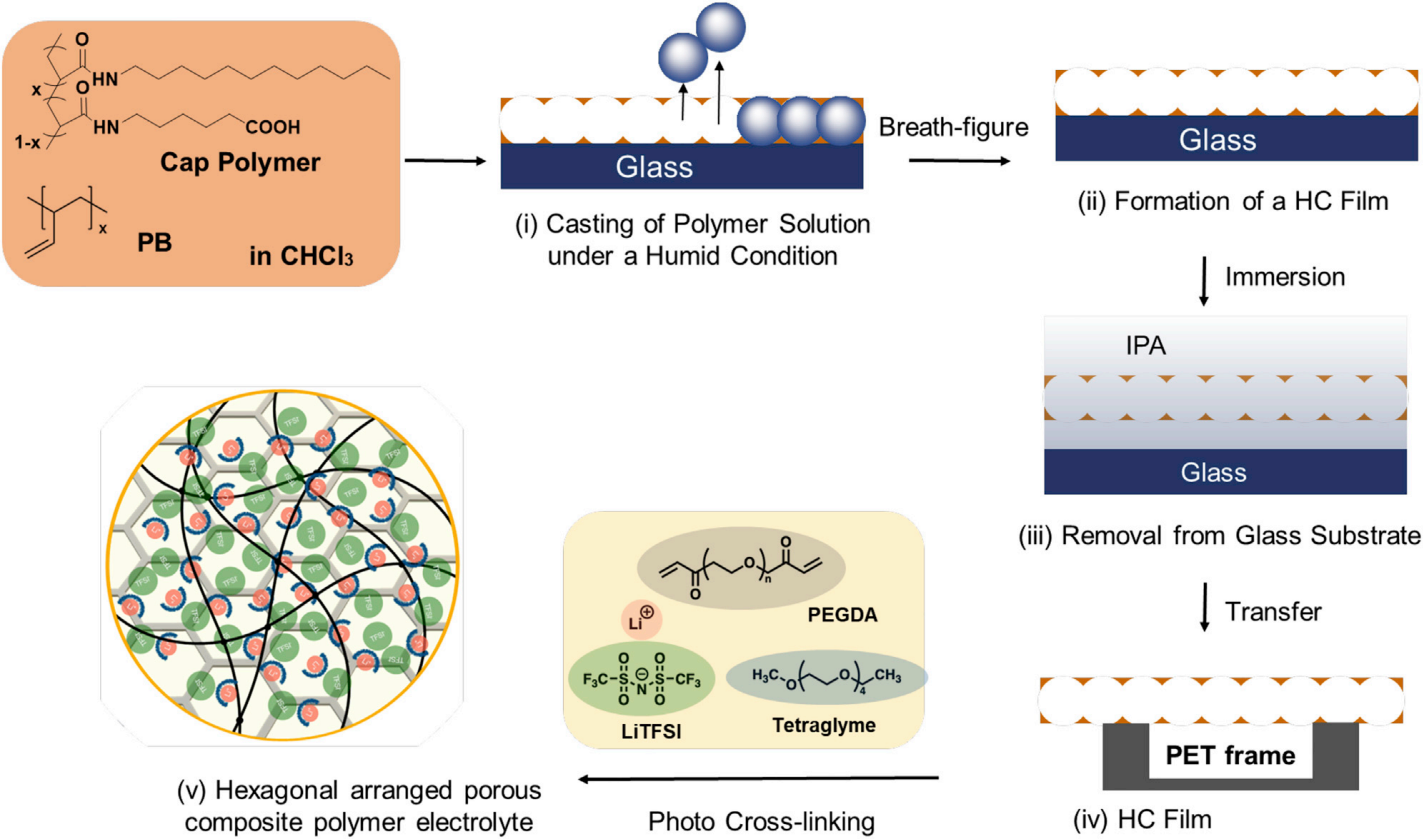
Schematics of the preparation of the HCP film and HCPE via photopolymerization under UV irradiation For a Figure360 author presentation of this figure, see https://doi.org/10.1016/j.isci.2022.104910.

In this work, we examine the application of porous hexagonal arranged porous polymer scaffolds with uniform pores as composite electrolytes for LIBs that can simultaneously provide high ionic conductivity, good processability, a wide electrochemical operating window, good thermal stability, good interfacial properties, and uniform lithium stripping and plating over many hours of operation. We used the breath-figure method to fabricate a robust HCP film containing poly(ethylene glycol) diacrylate (PEGDA) and equimolar ratios of tetraglyme (G4) and LiTFSI. Variants were prepared with different pore-sized hexagonal arranged porous films. The low molecular weight of PEGDA allowed complete mixing of G4 and LiTFSI with no additional solvent. A UV cross-linking reaction produced free-standing solid composite polymer electrolytes. The hexagonal arranged porous composite polymer electrolytes (HCPEs) exhibited impressive ionic conductivity (>0.1 mS cm^−1^) at 25°C. Electrochemical and thermal characterizations were conducted to determine the stability of the HCPEs. Electrochemical performance evaluation of the Li/HCPE/Li LIBs demonstrated uniform lithium stripping and plating patterns over many hours. The results showed that the performance of the HCPEs was better than that of previously reported poly(ethylene glycol) (PEG)-based electrolytes. This investigation indicates that HCPE membranes could be used for advanced LIBs that are safe, cheap, and have good performance minimizing the use of additional separators. To our best knowledge, this is the first report of the use of a hexagonal arranged porous film composite as a mechanical barrier to achieve high lithium ion transfer and prevent dendrite formation. A video (Figure 360) shows the summary of the presented work on porous composites the preparatory method of which is represented in Figure 1 and also described under STAR Methods.

## Results and discussion

### Structural characterization of hexagonal arranged porous composite polymer electrolytes

The HCPEs were prepared by *in situ* UV curing of a homogeneous precursor solution consisting of PEGDA, G4, LiTFSI, and a photoinitiator applied to hexagonal arranged porous scaffolds of different pore sizes. Schematics of HCPE preparation and the self-supporting solid HCPE film are shown in Figure 1.

**Figure 2:**
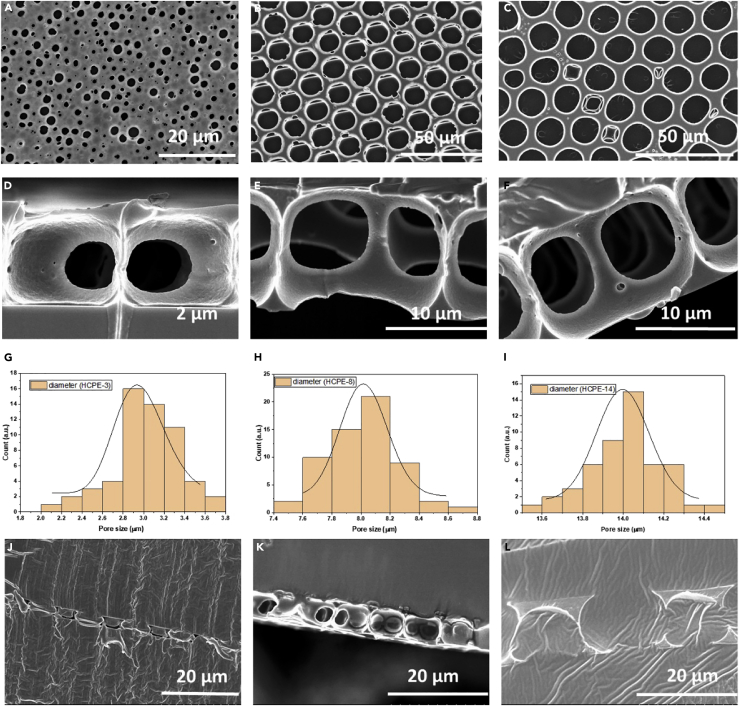
SEM images and properties of PB scaffolds SEM images of PB hexagonal arranged porous scaffolds with pore sizes of (A and D) 3 μm, (B and E) 8 μm, and (C and F) 14 μm, (G-I) corresponding pore size distributions, and SEM images of (J) HCP and HCPE-3, (K) HCPE-8, (L) and HCPE-14.

Figures 2A–2F show the SEM images and pore size distribution of pristine PB hexagonal arranged porous scaffolds prepared by the breath-figure method. The typical SEM images (top view, Figures 2A–2C and cross-section, Figures 2D–2F of the patterned PB films show regular morphology mimicking the HCP structure with average pore size distributions of 3, 8, and 14 μm. The size distributions of the pore sizes of the HCPE are shown in Figures 2G–2I. This type of porous network with a three-dimensional architecture with interconnected pores benefits rapid ion transfer into the interior pore channels of the composite electrolyte.

The SEM images in Figures 2J–2L show the wrinkled texture and highly compact structures after the pore channels or voids were filled with the polymer electrolyte matrix consisting of PEGDA, glyme, and LiTFSI. The wrinkled texture may have been caused by the cross-linking domains between PEG chains. The resulting HCPE membranes are flexible, which helps to achieve and maintain close contact between the electrolyte membrane and the electrodes. After preparing the composites and UV curing, the HCPE membrane became translucent, tack-free, and easy to manage. The cross-section shows that the polymer matrix was incorporated smoothly without disturbing the hexagonal arranged porous morphology. Overall, the SEM images suggest that the composite HCPE membranes had a uniform dispersion of the constituents, allowing the formation of a smooth, continuous network surface.

**Figure 3:**
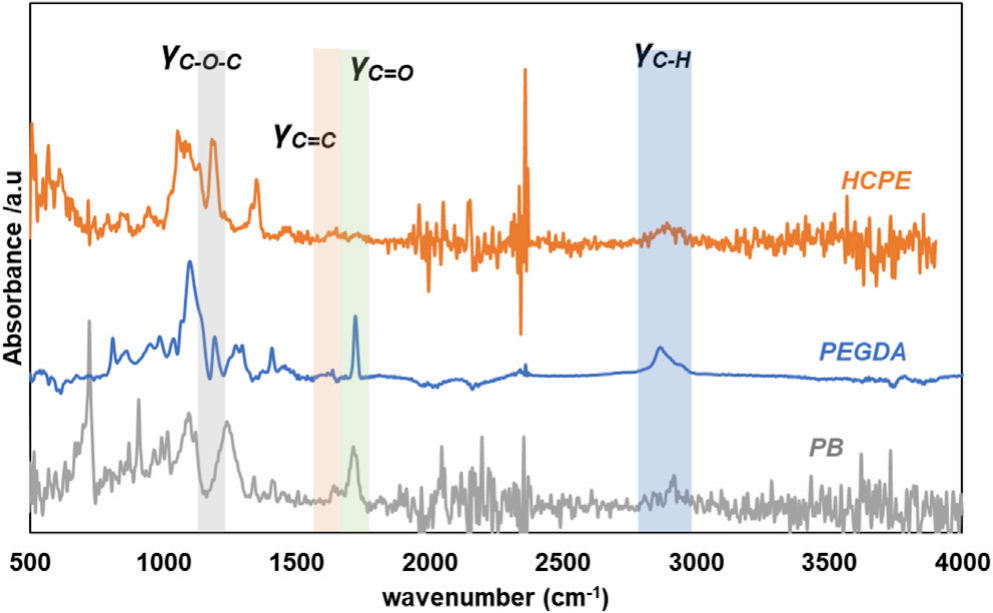
FT-IR spectra of PB, PEGDA, and HCPE composite electrolytes

FTIR was performed before and after UV curing to measure any differences in the chemical structure of HCPEs induced by UV irradiation (Figure 3). Precursor PEGDA showed characteristic -C=C- stretching at 1638 cm^−1^. The peaks in PB scaffolds at 1094 1240, 1408, and 1638 cm^−1^, are attributed to *δ*_C-C-H_ (out of plane), *δ*_C=C-H_ (out of plane), *δ*_C=C-H_ (in plane), and *ν*_C=C,_ respectively, were observed. The characteristic peak of the -C=C- (diacrylate) band at 1638 cm^−1^ diminished in the HCPE systems. The peak at 1725 cm^−1^ is attributed to *σ*_C=O_. The absorbance of the band at 3007 cm^−1^, corresponding to olefinic C-H stretching, increased at a much faster rate than those of the other C-H stretching signatures at 2960 and 2922 cm^−1^. The broad peak around 3500 cm^−1^ is attributed to -OH stretching of HCPE. No noticeable changes were observed, which confirmed that the hexagonal arranged porous patterns were intact and UV curing did not induce any major changes in the polymeric components or salt contents.

### Thermal analysis

**Figure 4:**
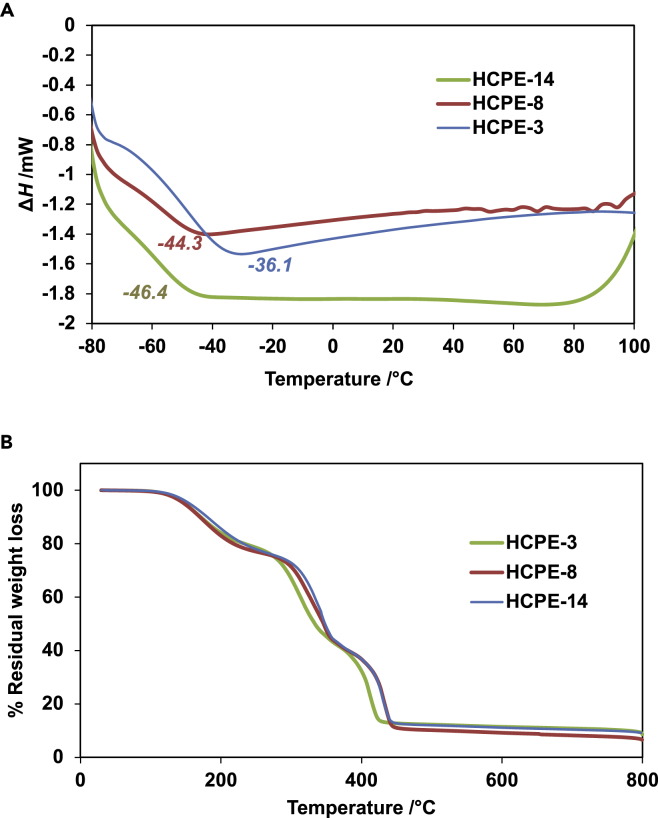
Thermal behavior of HCPE (A) DSC curves and (B) TGA profiles.

DSC experiments were conducted to determine the phase behaviors of HCPEs, which are highly dependent on the network structures (Figure 1). In a typical measurement, the samples were scanned from −80 to 100°C at a temperature ramping rate of 10°C min^−1^. Glass transition temperature (*T*_*g*_) was determined from the mid-point of the heat capacity change observed during the transition from the glassy to rubbery state in the DSC curve. Table 1 summarizes the glass transition values as obtained in DSC profiles shown in Figure 4A; and the residual weight losses of 5% (*T*_d5_) and 10% (*T*_d10_) as shown in Figure 4B. The *T*_*g*_ values varied from -46.4 to −36.1°C. The melting of HCPEs takes place around 120°C. Pre-polymeric host PEGDA4, 14 and PB scaffolds have melting temperatures of 16°C and 80°C, respectively; however, in the HCPE DSC curves, only glass transitions in the range of −80 to 100°C can be detected. This shows that PEGDA crystallinity was completely suppressed by the incorporation of SILs and network structures in hexagonal arranged porous scaffolds. The PEGDA chains were covalently attached to each other and their network structures were close to the ideal structures shown in Figure 1. There are few reported *T*_*g*_ values for PEG-based polymer electrolytes in the range of −30 to −20°C (Grewal et al., 2019a, 2019b) and the *T*_*g*_ values of our HCPEs being well below this range suggests that the chain mobility and ionic conductivity via the interconnected hexagonal arranged porous pores were better. TGA was used to determine the residual percentage weight loss and the thermal stability of HCPE. The temperature was measured from room temperature to 800°C at a rate of 10°C min^−1^ under a nitrogen atmosphere to measure the weight percentages of different components. HCPEs were stable up to 150°C without substantial deterioration, suggesting that the HCPE films could be used at elevated temperatures of 100°C. There were three main weight losses for all HCPEs (Figure 4B). The initial weight loss corresponding to the decomposition of G4, the second was related to PEG decomposition, and the third was associated with lithium salt decomposition (Grewal et al., 2019, 2021; Li et al., 2018). The residual weight losses of 5% (*T*_*d5*_) and 10% (*T*_*d10*_) typically occurred in the ranges of 148.3-155.4°C and 170.9 to 180.4°C, respectively (Table 1). Considering the experimental errors related to measurements and the sample preparation, the total weight loss was consistent with the HCPE composition. The HCPE films could serve as an alternative to common organic carbonate-based electrolytes (Arbizzani et al., 2011), which exhibit low thermal stability below 100°C, to provide a safer barrier against physical conditions at higher temperatures and minimize the use of additional separators.

**Table 1:** Summary of the thermal behavior of HCPE electrolytes and comparison with PEO-based electrolytes

Name	T_g_(°C)	T_d5_(°C)	T_d10_(°C)
HCPE-3	−36.1	148.3	170.9
HCPE-8	−44.3	147.7	170.0
HCPE-14	−46.4	155.4	180.4
PEGDA-G4-Li	−36.0	162.20	186.35
PEO	−30.0	333	350

Related to Figure 4.

### Ionic conductivity

**Figure 5:**
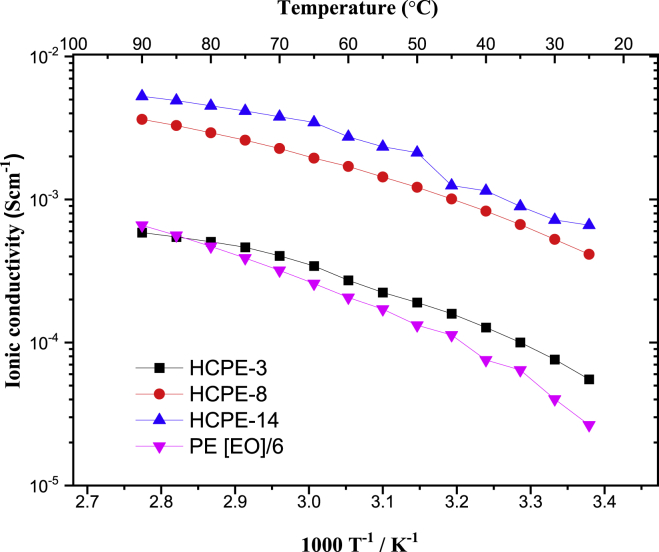
Temperature dependence of ionic conductivities of HCPEs

The ionic conductivity values of HCPEs were determined via AC impedance spectroscopy at temperatures between 25 and 90°C at an interval of 5°C Figure 5 shows the temperature-dependent ionic conductivities. Table 2 summarizes the calculated ionic conductivities at key temperatures (25, 60, and 90°C) which are shown in Figure 5 and calculated VTF parameters. HCPEs follow the simple Arrhenius model in the temperature range measured, in which ionic conductivities increase with increasing temperature. Typical values of HCPEs are in the range of 5.53 × 10^−5^ to 3.28 × 10^−4^ S cm^−1^ at 25°C, 2.72 × 10^−4^ to 2.74 × 10^−3^ S cm^−1^ at 60°C, and 5.86 × 10^−4^ to 5.27 × 10^−3^ S cm^−1^ at 90°C. These values are higher than those for polymer electrolytes without hexagonal arranged porous scaffolds (Grewal et al., 2020a, 2020b) (Table 2). Different HCPEs with different thicknesses (400-1500 μm), and among all the variants of HCPE films prepared, the ionic conductivities of HCPE-14 are the highest at a measured range of temperatures. Typical range of ionic conductivities of HCPEs are:

**Table 2:** Ionic conductivities and VTF parameters of HCPEs and comparison with previously reported electrolytes (ND: not determined; h: film thickness)

Sample	*h* (μm)	Ionic conductivity (Scm^−1^)			*E*_a_ (kJ mol^−1^)	Pre-factor, A	*T*_*0*_*(K)*	*R*^*2*^
		25°C	60°C	90°C				
HCPE-3	386.5	5.53 × 10^−5^	2.72 × 10^−4^	5.86 × 10^−4^	4.49	0.58	208.30	0.9975
HCPE-8	653.1	2.72 × 10^−4^	1.70 × 10^−3^	2.74 × 10^−3^	6.40	0.46	178.70	0.9999
HCPE-14	1461.7	3.28 × 10^−4^	2.74 × 10^−3^	5.27 × 10^−3^	5.07	0.54	197.65	0.9804
PEGDA-G4-Li	480.0	2.65 × 10^−5^	2.06 × 10^−4^	6.60 × 10^−4^	11.035	4.58	188.99	0.9997
PEO	200.0	5.25 × 10^−7^	3.49 × 10^−4^	1.49 × 10^−3^	ND	ND	ND	ND

Related to Figure 5.

HCPE-3: 1.33 × 10^−5^ to 5.53 × 10^−5^ S cm^−1^ at 25°C, 1.47 × 10^−4^ to 2.72 × 10^−4^ S cm^−1^ at 60°C, and 3.28 × 10^−4^ to 6.36 × 10^−4^ S cm^−1^ at 90°C. HCPE-8: 2.12 × 10^−5^ to 4.14 × 10^−4^ S cm^−1^ at 25°C, 1.70 × 10^−4^ to 1.06 × 10^−3^ S cm^−1^ at 60°C, and 5.75 × 10^−4^ to 3.63 × 10^−3^ S cm^−1^ at 90°C. HCPE-14: 3.34 × 10^−5^ to 6.66 × 10^−4^ S cm^−1^ at 25°C, 3.57 × 10^−4^ to 2.74 × 10^−3^ S cm^−1^ at 60°C, and 1.19 × 10^−3^ to 5.27 × 10^−3^ S cm^−1^ at 90°C. Summary of few HCPEs are shown in Table S2.

High ionic conductivities were obtained by eliminating the crystalline phase of the polymer and inducing LiTFSI salt dissociation owing to the interconnected pores, which allowed easy ion transfer. The diffusion scale of lithium ions in PEO is 1-100 μm (Orädd et al., 2002). In the present work, the hexagonal arranged porous film has pore sizes suitable for aiding ionic diffusion. Pores smaller than the micrometer scale can hinder ion transport by creating steric barriers or increasing internal resistance. Generally, HCPEs with a large pore size show high ionic conductivity owing to the high ionic mobility. By comparison, the usual porous separator PE membrane has low pore size uniformity, and the pore size range is completely different from those of hexagonal arranged porous films reported here. Other porous membranes may also not be suitable for rectifying the Li ion transport in terms of diffusion length. The size uniformity is crucial to evaluate the effect of pore size on ionic conductivity. Based on these reasons, hexagonal arranged porous composite film is one of the good candidates of porous materials to be composited with polymer electrolytes. Also, from the reported literature on porous materials (Kitazawa et al., 2014; Unemoto et al., 2013; Saito et al., 2007; Snedden et al., 2003; Chereddy et al., 2020). Aliahmad et al., 2016, the size of pores is less than 100 nm scale and non-uniform; therefore, the hexagonal arranged porous film may assist the rectification of ionic conductivity. A comparison figure (Figure S1) and table (Table S1) are included to ease in understanding of the diffusion of porous materials. Additional references are included to support Figure S1 and Table S1 (Lee et al., 2014; O’Dwyer, 2016; Yang et al., 2020). Figure S1 shows the Li-ion diffusion behavior of different porous materials while Table S1 summarized the size and size distribution of composited materials with solvate ionic liquids and compared them with the hexagonal porous materials reported in the present work and mentioned under STAR Methods.

Thermogravimetric analysis confirmed that PEGDA-supported HCPEs remained stable up to 150°C. The above-mentioned ionic trend was explained by the ionic conductivity activation energy (*E*_*a*_) obtained from the VTF equation (Equation 2) to clarify the underlying mechanism of ionic conductivity in HCPEs. The calculated thermodynamics and electronic parameters derived from VTF non-linear fits are shown in Table 2 along with uncertainties. *E*_*a*_ describes the relative strength of the interaction between the PEGDA hexagonal arranged porous scaffolds with varying pore sizes and SILs. *E*_*a*_ was calculated by fitting the values with the VTF equation. The corresponding plots (Figure S2, equations reported in STAR Methods) were used to determine the *E*_*a*_ of HCPEs. *E*_*a*_ was calculated to be in the range of 4.49-6.40 kJ mol^−1^ and it increased with increasing pore size. The values shown in the table are the overall range with uncertainty taken into account. The reason for such high tolerance is owing to the difference in thicknesses of the polymer membranes used. The authors prepared membranes multiple times in each same salt concentration condition. The obtained polymer membranes with different thicknesses, often occurred because the authors prepared the membranes on flat glass plates, not in Petri dishes with specific areas. The calculated activation energies and ionic conductivities show that the overall performance of HCPEs was better than those of previously reported PEO-based electrolytes (Grewal et al., 2019a, 2019b).

### Lithium-ion transference number of hexagonal arranged porous composite polymer electrolytes

**Figure 6:**
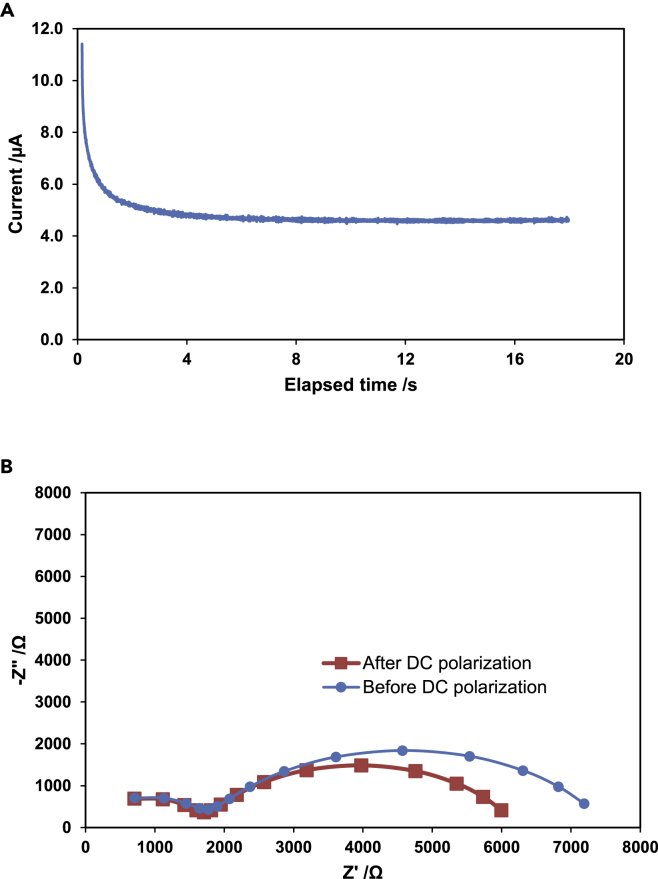
Electrochemical performances of the composite electrolytes (A) Polarization curve obtained by chronoamperometry for the Li/HCPE-8/Li symmetric cell at 60°C and (B) Nyquist plots for the HCPE symmetric cell.

Lithium-ion transfer number (*t*_Li+_) is also an important parameter for the electrolyte because a large lithium transference would suppress undesirable side reactions on the electrodes and would reduce electrode polarization (Kasnatscheew et al., 2021 (Materials Today) and Stolz et al., 2022). Low *t*_Li+_ may allow the ionic concentration to increase leading to high internal and interfacial resistances and reducing the overall electrochemical performance of LIBs. From the Bruce-VincentEvans equation (Equation 3), the *t*_Li+_ values of HCPEs were calculated as 0.32 to 0.40, which were much higher than those of PEO-based electrolytes (0.10-0.20). The high *t*_Li+_ values were attributed to the entrapment of large anionic species in the hexagonal arranged porous architecture and the cross-linked networks, allowing lithium ions to be the main conducting species. Figure 6 shows the corresponding graphs for a representative example of HCPE-8. Figure 6A shows the polarization curves obtained by chronoamperometry for the Li/HCPE-8/Li symmetric cell at 60°C, and Figure 6B shows the Nyquist plots of the symmetric cell before and after DC polarization.

### Electrochemical stability window

**Figure 7:**
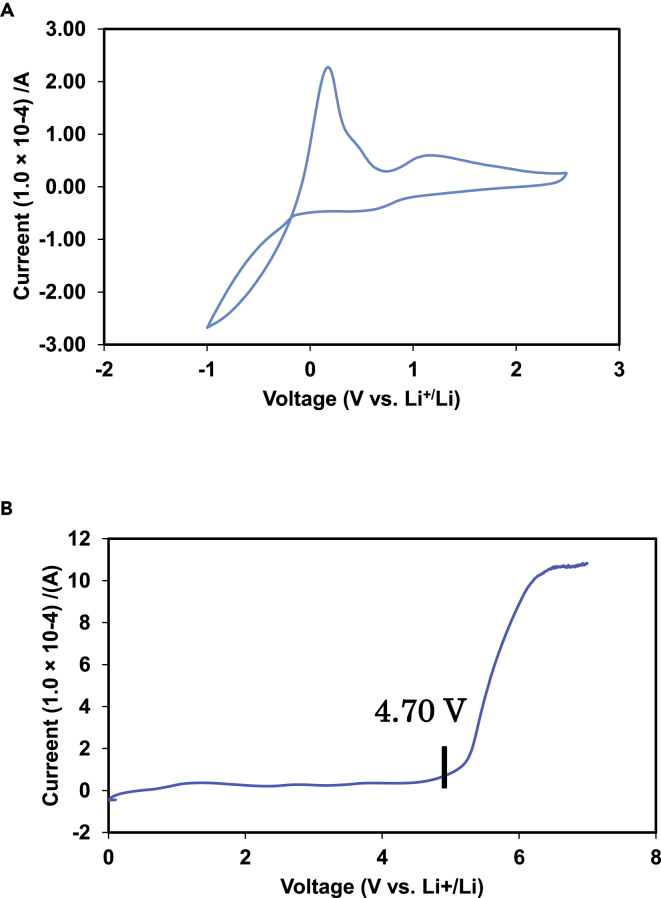
Coin cell performance (A) Cyclic voltammogram of a representative coin cell with a configuration of SUS304/HCPE-8/Li measured at a scan rate of 1 mV s^−1^ at 60°C. (B) LSV curve of the SUS304/HCPE-3/Li asymmetric cell.

The electrochemical stability window of an electrolyte is a fundamental parameter that determines the energy output and durability of rechargeable LIBs. The electrochemical stability of HCPEs was measured by CV and LSV in a cell with a configuration of stainless steel/HCPE/Li at 60°C Figure 7 shows the electrochemical stability of HCPE samples toward anodic oxidation and cathodic reduction reactions.

The cathodic profile (Figure 7A) showed the lithium stripping and plating processes. A reduction current was observed from −1.0C in the negative scan, which was owing to a lithium plating process on the stainless-steel electrode. In the following positive scan, a peak of 0.32 V was observed, corresponding to the lithium stripping process. The anodic stability was examined by LSV and Figure 7B shows that HCPE had an oxidation decomposition onset potential at 4.70 V, which indicates that HCPEs were stable up to 4.70 V (vs Li^+^/Li). This wide electrochemical window shows HCPEs can be used for 4 V conventional cathodes safely, a characteristic that is crucial for using HCPEs with high-voltage cathode materials. The presence of G4 in the composite electrolyte increases the overall oxidation stability compared with PEO electrolytes (4.5 V vs Li^+^/Li) (Wang et al., 2013; Park et al., 2003, Homann et al., 2020) owing to terminal methyl end groups, which prevent the interaction between the cathode surface and -OH terminal groups that occurs in PEO electrolytes.

### Lithium stripping and plating

**Figure 8:**
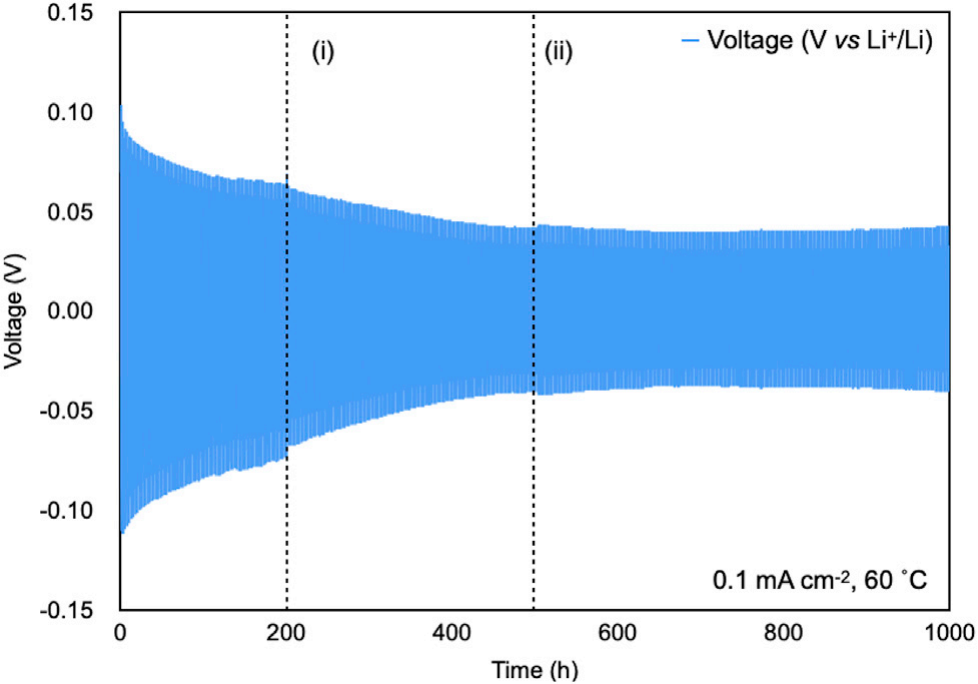
Lithium plating-stripping galvanostatic cycling of the Li/HCPE-8/Li symmetric cell cycled for 1000 h with a constant current of 0.1 mA cm^−2^ The cell impedance was measured at (i) 200 h and (ii) 500 h (dashed vertical lines).

The interface between the polymer electrolyte and electrode is important for the cycling performance in LIBs (Kido et al., 2015). To study the electrodeposition of lithium in HCPEs, galvanostatic cyclic experiments were conducted on the Li/HCPE/Li symmetric cells. Cells were charged and discharged at 60°C for 30 min at a current density of 0.1 mA cm^−2^ to mimic lithium stripping and plating. Typical galvanostatic cycling curves of the lithium symmetric cells obtained for HCPEs are shown in Figure 8. The current density was fixed, whereas the voltage of the cell was open, which became stable when the stable Li/HCPE interface layer was formed. The HCPE, which has a large porous area, remained in intimate contact with the lithium metal surface throughout repeated plating and stripping cycles and allowed easy penetration of lithium ions both within and between layers. Owing to the excellent interfacial compatibility of HCPE with lithium metal, the interfacial resistance remained low and constant throughout the measurements. It can be noted that the total charge carried during both lithium and stripping process is not very high; however, one can hypothesize that this leaves a good option for pursuing this path for future studies. We believe that the hexagonal arranged porous scaffolds and cross-linked structures allow the migration of lithium ions, but limit the mobility of lithium clusters which can lead to lithium dendrites. Apart from the relatively high voltage polarization initially for electrode activation and formation of solid electrolyte interphase (SEI) layer, the plating, and stripping of Li metal in Li/HCPE/Li cells occurred at a low overpotential around 5.0 mV. Evidently, when the cell is cycled for over 1000 h it maintains low and stable voltage polarization without short circuiting, demonstrating highly stable lithium stripping/plating processes. The symmetric cell maintained smooth deposition and dissolution of lithium, and hence a stable voltage polarization without short circuiting, even after prolonged working conditions of 1000 h. The time evolution of the impedance spectra of the symmetric lithium coin cell under open circuit conditions also demonstrated the electrochemical stability of HCPE (Figure S3). The magnified profiles for 90-100 hours are shown in supplemental.

Furthermore, to investigate the feasibility of HCPE, a cell with configuration LiFePO4 | HCPE-8 | Li metal was constructed for the lithium-ion battery test. The corresponding charge-discharge profiles of the constructed cell at 0.01C under 60°C are shown in Figure S4. The initial discharge capacity of the LFP | HCPE-8 | Li cell at 0.01C was 77 mAh g^−1^ which is much smaller than the theoretical capacity of LiFePO_4_ (170 mAH g^−1^), and the discharge capacity further decreased gradually. For comparison with PEG-based electrolytes, the discharge HCPE-8 is slightly better than that of poly(PEGDA-PEMP) and PEO (previously reported, Grewal et al, 2019a) although still lower than the theoretical capacity. The low discharge capacities show the active cathode material has not been fully utilized in the cathode. Experiments to optimize better cathode materials and improve overall battery performance are in progress. Once optimized, the results will be published elsewhere.

These are simply the preliminary results as the battery performance test was carried out using commercially available cathode material. Experiments to optimize better cathode materials and improve battery performance are in progress. Efforts to improve electrode/electrolyte interface structure are in progress. One way to improve the battery performance is by constructing integrated cathode material with the HCPEs and comparing the performance.

In summary, PEG electrolytes containing SILs are promising polymer electrolyte candidates for developing high-performance LIBs; however, these polymer electrolytes (molten state) must still be contained to prevent leakage and ensure smooth electrochemical performance. Incorporating polymer electrolytes into hexagonal arranged porous scaffolds containing interconnected pores that ensure smooth ion conduction may prevent leakage. The three-dimensional hierarchical porous architecture of hexagonal arranged porous scaffolds reduces the ion diffusion resistance and promotes rapid ion transfer into the internal pore sites. Additionally, the high surface area would increase the accumulation of electrolyte ions at the interface of the electrode/composite electrolyte. Therefore, we proposed an HCPE composed of PEGDA and SILs to achieve high ionic conductivity, high thermal stability, and a wide potential window simultaneously. We used the breath-figure technique to prepare hexagonal arranged porous scaffolds with a unique porous architecture containing interconnected pores to provide an optimum route for ion transfer. The HCPEs exhibited a high ionic conductivity and attractive electrochemical and mechanical properties, including a high lithium transference number. The thermal and electrochemical properties of the composite polymer electrolytes were strongly affected by the SIL concentration, and maintaining their concentration contributed to the thermal stability and the oxidation stability of more than 4.5 V vs Li/Li^+^. The integration of hexagonal arranged porous scaffolds with uniform pores in composite electrolytes for LIBs can simultaneously provide high ionic conductivity, good processability, a wide electrochemical operating window, and good thermal stability. Our study provides insights into the optimal design of multifunctional electrolytes for energy storage systems. Next, we intend to test the cyclability and rate capability of all-solid-state batteries containing HCPEs at ambient temperature. Overall, the present work shows that HCPEs are promising candidates for avoiding the formation of crystalline PEO and that the hexagonal arranged porous scaffold is suitable as a mechanical barrier to achieve high lithium-ion transfer and prevent dendrite formation.

### Data availability

All data are available in the main text and supplemental information.

### Limitations of the study

In this study, the hexagonal arranged porous films were prepared under carefully optimized concentrations of constituting materials and physical conditions of temperature and humidity. Detailed studies related to battery profiles (charge and discharge) with different cathode materials at different current densities were not carried out in detail and should be of interest for further studies. SEM studies related to lithium dendrites were not carried out in the present study. Furthermore, we believe extensive computational and experimental works are necessary to understand the underlying mechanisms for improved electrochemical performance of such composite electrolytes.

## STAR★Methods

### Key resources table

**Table:** 

REAGENT or MATERIALS	SOURCE	IDENTIFIER
Polybutadiene (RB820)	JSR, Tokyo, Japan	n/a
Chloroform	Sigma-Aldrich (St. Louis, MO), USA; Fujifilm Wako Chemicals, Osaka, Japan	67-66-3, 1731042
Polyethylene glycol diacrylate (PEGDA 700)	Sigma-Aldrich (St. Louis, MO), USA	Mol. Wt. 700, 26570-48-9, 455008
Tetraethylene glycol dimethyl ether (Tetraglyme, G4)	Sigma-Aldrich (St. Louis, MO), USA	143-24-8, 1760005
2,2-dimethoxy-2-phenylacetophenone (DMAP)	Kanto Chemical Co., Inc., Tokyo	24650-42-8
Isopropanol	Fujifilm Wako Chemicals, Osaka, Japan	67-63-0
Battery fabrication components (coin cell (2032) parts and LiFePO4 cathodes)	Hohsen Corp., Osaka, Japan	n/a
Lithium metal	Honjo Metal Co., Ltd., Osaka, Japan	n/a
PET frame SAN1260	Sanplatec, Osaka, Japan	n/a

**Instrument and materials**		

UV lamp	Sunhayato, Tokyo, Japan	Chibi Light DX BOX-S1100
Glove box	MIWA, Ibaraki, Japan	n/a
Optical microscope	VHX-500, Keyence, Osaka, Japan	n/a
field-emission scanning electron microscopy (SEM; S-5200)	Hitachi, Tokyo, Japan	n/a
Osmium sputterer (HPC-1SW, Vacuum Device)	Ibaraki, Japan	n/a
Fourier-transform infrared (FT-IR) spectrometer (FT/IR-6100)	Jasco, Japan	n/a
TG-DTA (Thermo plus EvoII TG-DTA8210)	Rigaku, Tokyo, Japan	n/a
DSC (DSC 3)	Mettler Toledo, Greifensee, Switzerland	n/a
stainless-steel electrodes (SUS304)	Nilaco, Tokyo, Japan	n/a
AC impedance spectrometer (3532-80 LCR HiTester)	Hioki, Nagano, Japan	n/a
potentiostat/galvanostat (1470E)	Solartron Analytical, Farnborough, UK	n/a

**Software**		

Origin Pro 2020	n/a	n/a

### Resource availability

#### Lead contact

Further information and requests for resources and reagents (fabrication details of porous scaffolds and electrolytes preparation) should be directed to and will be fulfilled by the lead contact, Hiroshi Yabu (hiroshi.yabu.d5@tohoku.ac.jp).

#### Materials availability

This study did not generate new unique reagents.

### Experimental model and subject details

Polybutadiene (PB) (RB820) was kindly provided by JSR (Tokyo, Japan), and chloroform was purchased from Sigma-Aldrich (St. Louis, MO) and Fujifilm Wako Chemicals (Osaka, Japan). An amphiphilic copolymer (polymer 1, *Mw* = 40 kg mol^−1^) was synthesized according to a previously reported method (Kojima et al., 2009). Additionally, PEGDA (average *Mn* = 700, Sigma-Aldrich), G4 (Sigma-Aldrich), 2,2-dimethoxy-2-phenylacetophenone (DMPA; Sigma-Aldrich), and LiTFSI (Kanto Chemical Co., Inc., Tokyo) as a lithium salt were purchased. All reagents were used as received, with no further purification. A UV lamp (Chibi Light DX BOX-S1100, Sunhayato, Tokyo, Japan) was used for photopolymerization. Battery fabrication components (coin cell (2032) parts and LiFePO_4_ cathodes) were purchased from Hohsen Corp. (Osaka, Japan) and lithium metal was purchased from Honjo Metal Co., Ltd. (Osaka, Japan). Coin cells were assembled in argon-filled glove box (MIWA, Ibaraki City; O_2_ and H_2_O < 0.1 ppm).

### Method details

#### Fabrication of robust PB based HCP films using breath figure method

HCP films were prepared by depositing a chloroform solution (10–20 mL, 1–2 g L ^−1^) of PB and polymer 1 (weight ratio PB/polymer 1 = 10:1) on a glass substrate (10 × 30 cm^2^) and placing it in a closed chamber under humid conditions (relative humidity ≈90%, velocity 130 L min^−1^) at ambient temperature (Figure 1). The surface of the solution became turbid due to the condensation of water droplets induced by the evaporative cooling effect of the volatile solvent, chloroform. A translucent white film was obtained after complete evaporation of chloroform and the water droplets. Figure 1 shows a schematic of the fabrication of the HCP films using the breath-figure technique. By adjusting the physical parameters, relative humidity, velocity, and molar concentration of the polymer solutions, HCP films with pore sizes of 3, 8, and 14 μm were prepared, and were named HCP-3, HCP-8, and HCP-14, respectively. The films were removed using isopropanol (Fujifilm Wako Chemicals) and tweezers and transferred onto a PET frame (SAN1260, Sanplatec, Osaka, Japan).

#### Fabrication of hexagonal arranged porous composite polymer electrolytes (HCPE)

First, PEGDA (1.00 mL, 1.43 mmol, [EO] = 18.59 mmol), G4 (0.68 mL, 3.10 mmol), and LiTFSI (0.88 g, 3.10 mmol, where the molar ratio of the ether oxygen [EO] in PEGDA per lithium ion [Li] was [EO]/[Li] = 6) were mixed in a glass vial and stirred for 24 h. Before photopolymerization, a catalytic amount of DMPA was added to the solution as a photoinitiator. All precursor solutions were visually confirmed to be well-mixed and homogeneous. The resultant solution was poured onto the HCP-3, HCP-8, and HCP-14 films and photoirradiated with the UV lamp at 356 nm from front and back for 5 min each to obtain the solid composite HCPEs, HCPE-3, HCPE-8, and HCPE-14, respectively.

### Experimental model and subject details

The HCP films were examined by optical microscopy (VHX-500, Keyence, Osaka, Japan) and field-emission scanning electron microscopy (SEM; S-5200, Hitachi, Tokyo, Japan) after the specimens were coated with Os using an osmium sputterer (HPC-1SW, Vacuum Device, Ibaraki, Japan). The HCPE structures were characterized before and after curing by Fourier transform infrared (FT-IR) spectroscopy (FT/IR-6100, JASCO; equipped with a diamond-attenuated total reflection accessory) between 4000 and 600 cm^−1^. The morphology of the HCPEs was observed by SEM (S-5200, Hitachi). Small parts of the HCPE specimens were stuck to a copper grid with double-sided adhesive conductive carbon tape and sputtered with osmium (HPC-1SW, Vacuum Device) to minimize charging problems. Pore size distributions were plotted as histograms in Origin Pro (2020). The thermal properties of the HCPEs were determined by thermogravimetric-differential thermal analysis (TG-DTA) and differential scanning calorimetry (DSC) analysis. TG-DTA (Thermo plus EvoII TG-DTA8210, Rigaku, Tokyo, Japan) was performed to determine the weight percentage and thermal stability, whereas DSC (DSC 3, Mettler Toledo, Greifensee, Switzerland) was performed to determine the glass transition temperature and crystallinity of the polymer electrolytes. Samples were measured at a 10°C min^−1^ ramp rate under a nitrogen atmosphere. The polymer specimens were dried at 60°C overnight under vacuum to remove any absorbed moisture prior to TG-DTA and DSC measurements.

#### Electrochemical characterization

The ionic conductivity of HCPE films (round punched, 16 mm diameter) was measured by electrochemical impedance spectroscopy of coin cells (2032), in which HCPEs were sandwiched between two stainless-steel electrodes (SUS304, Nilaco, Tokyo, Japan). AC impedance spectroscopy (3532-80 LCR HiTester, Hioki, Nagano, Japan) measurements were carried out in a frequency range of 50 Hz to 5 MHz with a perturbation voltage of 5 mV and a temperature range of 25–90°C with a temperature interval of 5°C. To ensure thermal equilibration and data reproducibility, a 30 min holding time was allowed between each temperature step. The ionic conductivity was then calculated as

(Equation 1) $$\sigma =l/\left(R_{b}\times A\right)$$

where *σ* is the ionic conductivity (S cm^−1^), *l* is the thickness (cm), *A* is the contact area (cm^2^), and *R*_*b*_ (Ω) is the bulk resistance of the HCPE membrane. *R*_*b*_ was obtained from the Nyquist plot of complex impedance measurements. To fit the behavior of the temperature-dependent ionic-conductivities of HCPEs, we used the Vogel-Tamman-Fulcher (VTF) equation,

(Equation 2) $$\sigma_{1}=\sigma_{0}\times {\text{T}}^{-1/2}\;\exp\left[-E_{a}/\left\{R\left(T-T_{0}\right)\right\}\right]$$

where *σ*_1_ is ionic conductivity at temperature T, *σ*_0_ is the pre-exponential factor, *E*_*a*_ is the activation energy for lithium-ion conduction, *R* is the universal gas constant, and *T*_*0*_ is the Vogel temperature, which is equal to the glass transition temperature in ideal gases.

The electrochemical stabilities of the HCPEs were evaluated using cyclic voltammetry (CV) and linear sweep voltammetry (LSV) using a potentiostat/galvanostat (1470E, Solartron Analytical, Farnborough, UK) with a cell configuration of stainless steel (SUS304, Nilaco)/HCPE/Li in the scan range of - 0.1 to 7 V (vs Li+/Li) at 60°C. Separately, the scan ranges for CV and LSV were from −1.5 to +1.5 V and from 1.0 to 7.0 V, respectively. The scan rate was 1 mV/s for both CV and LSV. Lithium plating/stripping cycling was conducted with a Li/HCPE/Li cell at a current density of 0.1 mA cm^−2^ using a battery tester (580 Battery Test System, Scribner Associates, Southern Pines, NC). Interfacial resistances were measured using a frequency response analyzer (1470E, Solartron Analytical) after certain intervals during lithium stripping and plating cycling. The lithium transference number*, t*_Li+_, was determined by a combination of electrochemical impedance spectroscopy and potential polarization using cell assemblies with a configuration of Li/HCPE/Li. After stabilization at 60°C overnight, a DC potential of 10 mV was applied until a steady current was obtained, and the initial (*I*_0_) and steady (*I*_s_) currents were measured. Simultaneously, initial (*R*_0_) and steady-state interfacial (*R*_s_) resistances were measured. The *t*_Li+_ values were obtained from the Bruce-Vincent-Evans equation,

(Equation 3) $$t_{\text{Li}+}=\frac{Is\;\left(\Delta V-I0R0\right)}{I0\;\left(\Delta V-IsRs\right)}$$

### Quantification and statistical analysis

Details of statistical analysis are provided within the relevant figure legends, their legends, and associated detailed methods.

Pore size distribution were represented as histograms and plotted in Origin Pro (2020). The histogram is produced by splitting the data range into bins of equal size. Bins are the class range (lower boundary bin < upper boundary) for frequency counts. Then for each bin, the number of points from the dataset that fall into each bin is counted. The result is a plot of frequency (i.e. counts for each bin) on the vertical axis vs. response variable (pore size, μm) on the horizontal axis.

## Data Availability

• This paper does not report original code. • Any additional information required to reanalyze the data reported in this paper is available from the lead contact upon request.

## Supplemental information

Document S1. Figures S1–S6, Tables S1 and S2

Figure 360. An author representation of Figure 1
