# Supplementary material for: Increasing the ionic conductivity and lithium-ion transport of photo-cross-linked polymer with hexagonal arranged porous film hybrids
Source: iScience. 2022 Aug 13;25(9):104910. doi: 10.1016/j.isci.2022.104910 (PMC9442354; doi:10.1016/j.isci.2022.104910)
Supplement: Document S1. Figures S1–S6, Tables S1 and S2 [file mmc1.pdf]

iScience, Volume *25*

Supplemental information

Increasing the ionic conductivity and lithium-ion transport of photo-cross-linked polymer with hexagonal arranged porous film hybrids

Manjit Singh Grewal, Kazuaki Kisu, Shin-ichi Orimo, and Hiroshi Yabu

Supplementary Information

Increasing the Ionic conductivity and Lithium-Ion Transport of Photo-Cross-Linked Polymer Electrolytes with Hexagonal Arranged Porous Film Hybrids

Manjit Singh Grewal[1,*], Kazuaki Kisu[2], Shin-ichi Orimo[1, 2], and Hiroshi Yabu[1, 3, 4, *]

[1]Advanced Institute of Materials Research (WPI-AIMR), Tohoku University, 2-1-1, Katahira, Aoba-Ku, Sendai 980-8577, Japan; [2]Institute for Materials Research (IMR), Tohoku University, Katahira 2-1-1, Aoba-ku, Sendai, 980-8577, Japan; [3]Institute of Multidisciplinary Research for Advanced Materials (IMRAM), Tohoku University, 2-1-1, Katahira, Aoba-Ku, Sendai 980-8577, Japan; [4]RIKEN Center for Emergent Matter Science, 2-1, Hirosawa, Wako, Saitama 351-0198, Japan, TEL/FAX: ＋81-22-217-6341/634.

*Correspondence: grewal.manjit.singh.d3@tohoku.ac.jp, hiroshi.yabu.d5@tohoku.ac.jp

Lead contact

Further information and requests for resources and reagents (fabrication details of porous scaffolds and electrolytes preparation) should be directed to and will be fulfilled by the lead contact, Hiroshi Yabu (**hiroshi.yabu.d5@tohoku.ac.jp**).

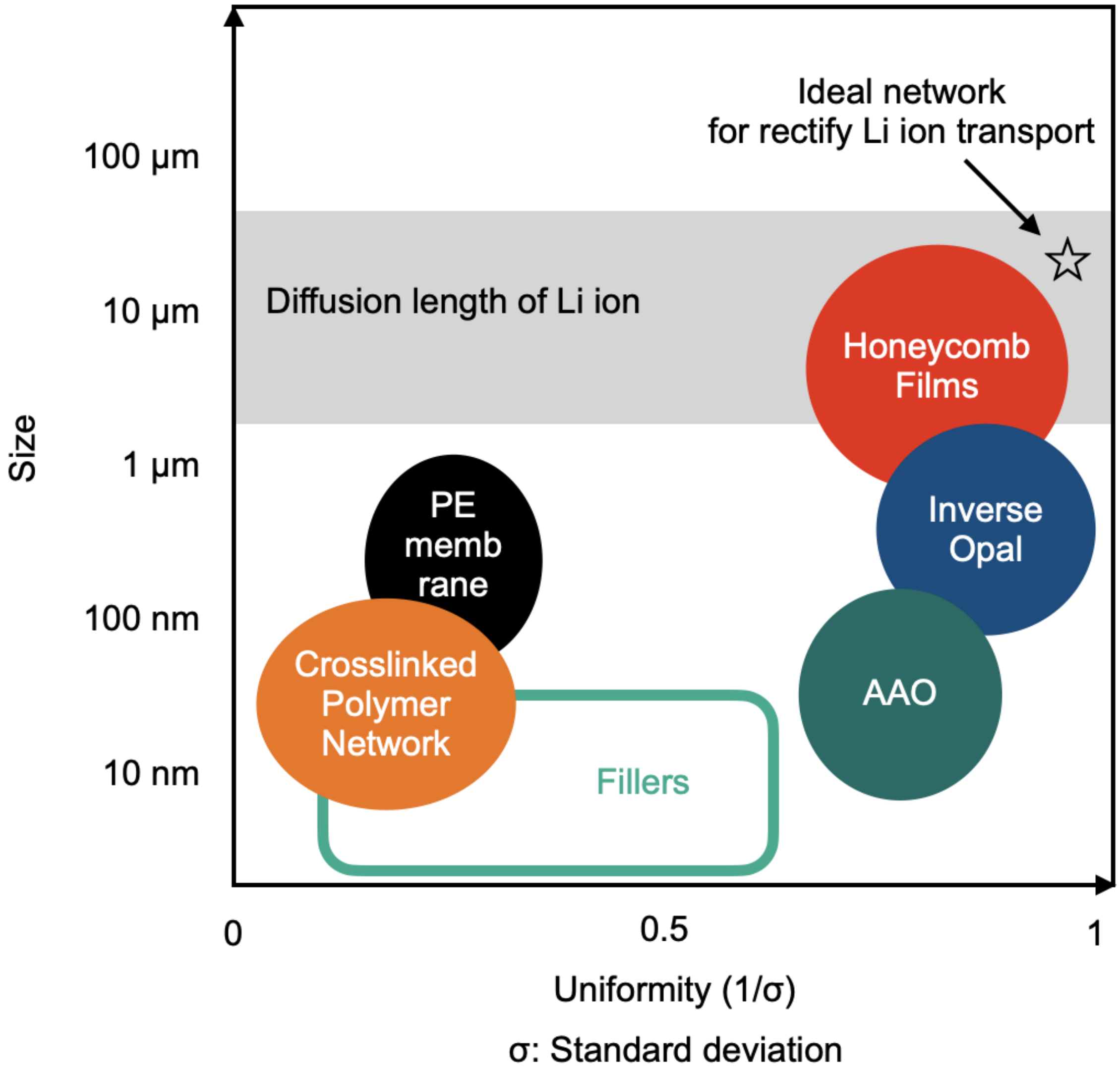

Figure S1: Comparison of diffusion behaviour of different porous materials, Related to Figure 1.

Table S1: Summary of size and size distribution of composited materials with solvate ionic liquids, Related to Figure 1 and STAR methods.

	**Sample**	**Average Size**	**Size Distribution**	**Literature**
Polymer Networks	Cross-linked polymer network	10-100 nm	10-1000 nm	Macromolecules 2003;36(12);4549–4556.
Fillers	PSt cores and PMMA loop coronas	20 nm	10-40 nm	Macromolecules 2014;47;6009-6016.
	Silica	7 nm	σ >20 %	J. Phys. Chem. B 2007;111;11794-11802.
	PVDF-HFP	1 µm	σ >20 %	AIP Advances 2016;6;065206.
	Cellulose nanofibrils (CNFs)	30–40 nm	σ >20 %	Energy Storage Materials 2020;28;293–299.
Porous Films (Separators)	PE membranes	0.01–0.50 µm	110-150 nm	Journal of Solid State Electrochemistry, 2014, 18, 2451–2458.
	Inverse opal	200-800 nm	σ <20 %	Adv.Mater.2016, 28, 5681–5688
	Anodic aluminium oxide (AAO)	70 nm	σ <20 %	J. Mater. Chem. A, 2020,8, 5095-5104
	Hexagonal arranged porous films	100 nm - 50 µm	σ <20 %	This work

σ : standard deviation values of pore sizes.

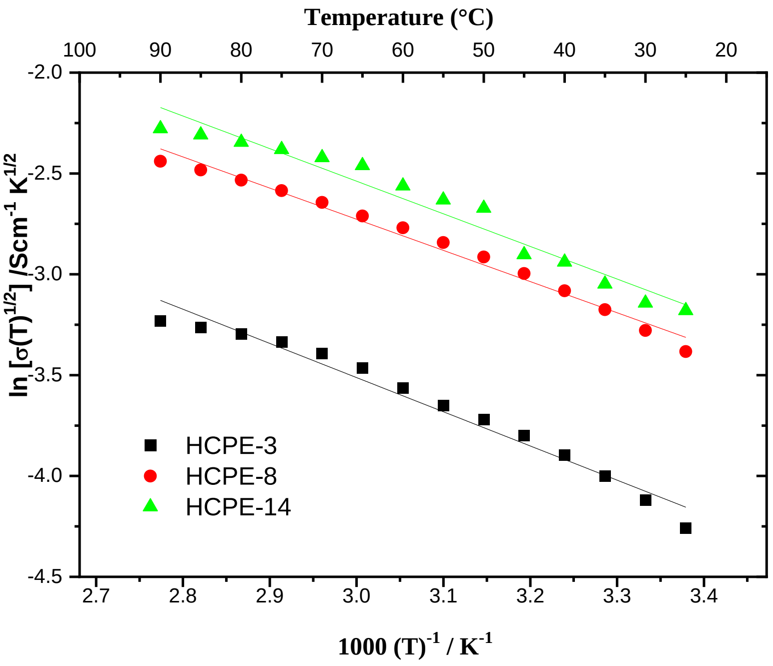

Figure S2: VTF fitting of HCPEs, related to Figure 5.

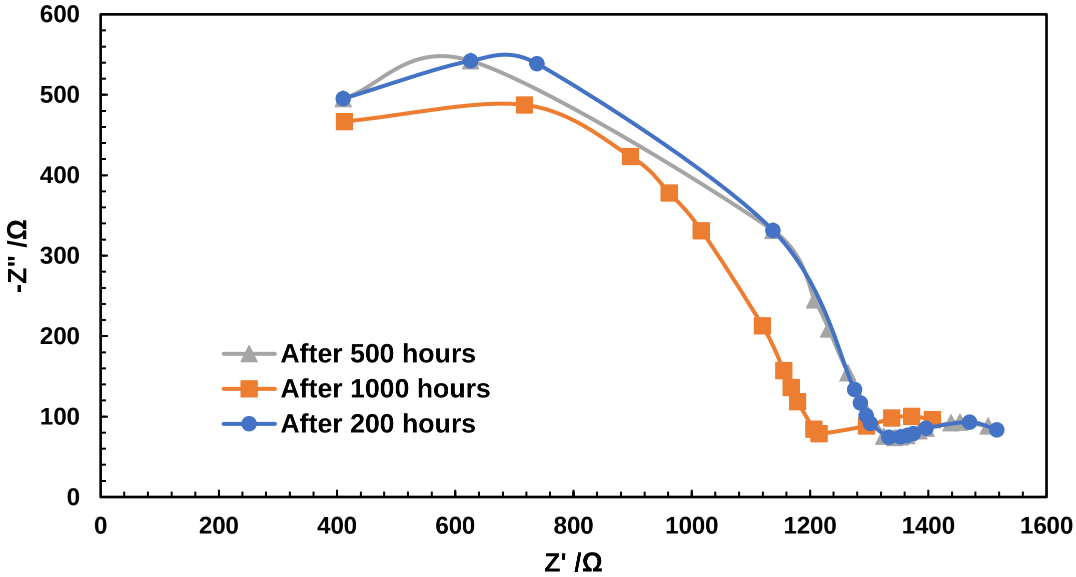

Figure S3: Time dependent interface impedance Li/HCPE-8/Li symmetric cell, Related to Figure 8.

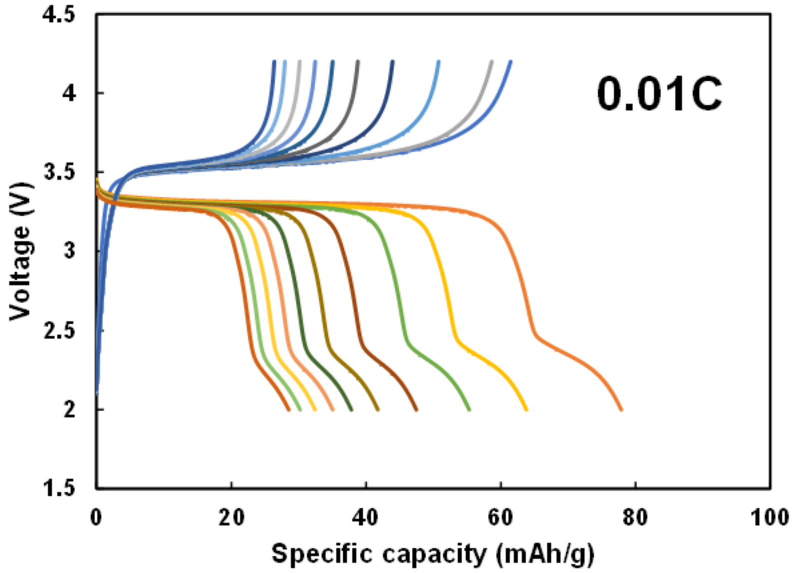

Figure S4: Charge/discharge plots of a representative coin cell assembled with the configuration of $LiFePO_4$ | HCPE-8 | Li at 0.01C rate at 60 °C, Related to Figure 8 and STAR methods.

Table S2: Ionic conductivities of HCPEs, Related to Table 1.

Sample	Thickness of film (µm)	Ionic conductivity (Scm^{-1})		
		25 °C	60 °C	90 °C
**HCPE-3**	1089.6	1.33×10^{-5}	1.47×10^{-4}	5.64×10^{-4}
**HCPE-3**	1190.5	1.36×10^{-5}	1.66×10^{-4}	6.11×10^{-4}
**HCPE-3**	1099.5	1.45×10^{-5}	1.76×10^{-4}	6.36×10^{-4}
**HCPE-8**	1292.6	2.46×10^{-5}	2.62×10^{-4}	8.72×10^{-4}
**HCPE-8**	574.8	2.12×10^{-5}	1.84×10^{-4}	5.75×10^{-4}
**HCPE-8**	1412.8	5.25×10^{-5}	4.34×10^{-4}	1.29×10^{-3}
**HCPE-14**	918.9	3.34×10^{-5}	3.57×10^{-4}	1.19×10^{-3}
**HCPE-14**	1496.0	2.25×10^{-4}	1.06×10^{-3}	2.39×10^{-3}

**HCPE-14**	1461.7	6.66×10^{-4}	2.74×10^{-3}	5.27×10^{-3}

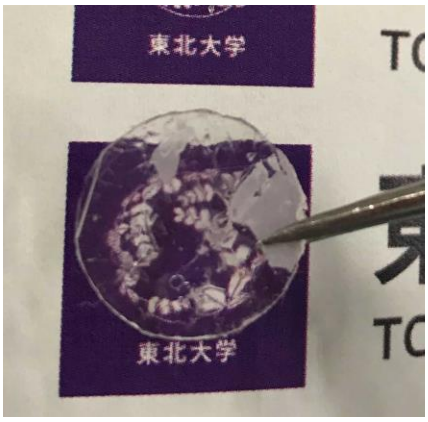

Figure S5: Photograph of HCPE, Related to Figure 1 and STAR methods.

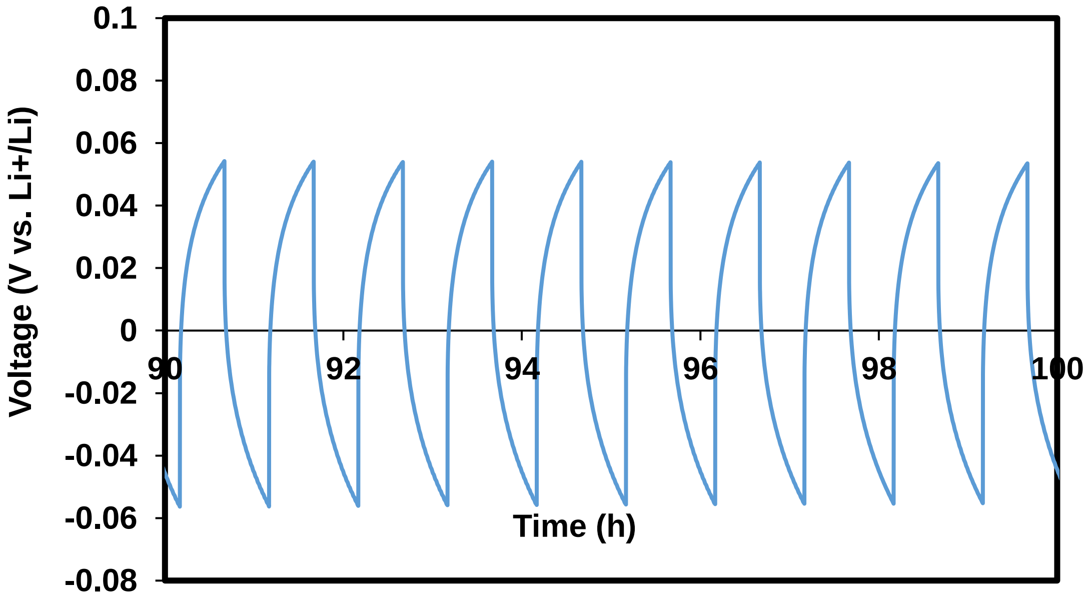

Figure S6: The lithium stripping and plating profiles for 90-100 hours, Related to Figure 8.
